# Comprehensive analysis about prognostic and immunological role of WTAP in pan-cancer

**DOI:** 10.3389/fgene.2022.1007696

**Published:** 2022-09-06

**Authors:** Jiangchu Lei, Yuzhi Fan, Chaobiao Yan, Yeernaer Jiamaliding, Yang Tang, Jiawei Zhou, Mengna Huang, Guomin Ju, Jian Wu, Chuanhui Peng

**Affiliations:** Division of Hepatobiliary and Pancreatic Surgery, Department of Surgery, First Affiliated Hospital, School of Medicine, Zhejiang University, Hangzhou, China

**Keywords:** WTAP, pan-cancer, database, survival analaysis, immune infiltration

## Abstract

**Background:** Wilms tumor 1-associated protein (WTAP) plays a critical role in ribonucleic acid (RNA) methylation of N6 adenosine (m^6^A) modification, which is closely related with varieties of biological process. However, the role of WTAP in cancers remains to be determined. This study is designed to demonstrate the prognostic landscape of WTAP in pan-cancer and explore the relationship between WTAP expression and immune infiltration.

**Methods:** Here, we investigated the expression level and prognostic role of WTAP in pan-cancer using multiple databases, including PrognoScan, GEPIA, and Kaplan-Meier Plotter. Then, applying the GEPIA and TIMER databases, we illustrated the correlations between WTAP expression and immune infiltration in tumors, especially liver hepatocellular carcinoma (LIHC), and esophageal carcinoma (ESCA).

**Results:** WTAP had significant higher expression levels in tumor tissues of ESCA, LIHC, etc., while lower expression levels in those of bladder urothelial carcinoma (BLCA), breast invasive carcinoma (BRCA), etc. And WTAP demonstrated multifaceted prognostic value in cancers. Of our interests, WTAP exerted a harmful effect on LIHC patient for overall survival (OS) and progression free survival (PFS). WTAP expression also significantly associated with the infiltration levels of B cells, CD8^+^ T cells, CD4^+^ T cells, macrophages, neutrophils, and dendritic cells (DC) in LIHC but not ESCA. Furthermore, combined analysis about WTAP expression level and immune cell specific gene markers implied WTAP correlates with regulatory cells (T reg) infiltration in LIHC and ESCA.

**Conclusion:** The m^6^A regulator WTAP can serve as a prognostic biomarker for certain tumor types in pan-cancer and potentially result from immune cell infiltration.

## Introduction

N6-methyladenosine (m^6^A) is the most abundant endocellular messenger RNA (mRNA) modification, which plays a significant role in almost every aspect of the mRNA transcription (Roundtree et al., 2017). m^6^A is installed co-transcriptionally by a complex called transmethylase consisting of METTL3, METTL14, and WTAP. Wilms tumor 1-associated protein (WTAP) is the consisting part that our research focuses on (Deepak et al., 2017; Wang et al., 2020).

There is an increasing body of literature that recognizes the expression level of WTAP is associated with progression and prognosis of cancers. It is worth noting that the incidence of various cancers does not simply show a certain trend with the level of WTAP expression, but shows specific effects for different cancer groups, such as for liver cancer (Chen et al., 2019; Zhou et al., 2021), bladder cancer (Chen and Wang, 2018; Wenjie et al., 2021), breast cancer (Huang et al., 2021; Ou et al., 2021; Wang et al., 2022) and many other ones, the high WTAP expression level is a risk factor. That is to say, with the increase of expression, the incidence of tumors increases significantly. While for lung cancer (Li et al., 2020a; Gu et al., 2021), melanoma (Feng et al., 2021), the result is exactly the opposite, high expression of WTAP is a protective factor. With the high expression of WTAP, the incidence of tumors decreases. But for some other cancers, contradicting conclusions were drawn in different studies, for instance, kidney cancer. He et al. (2021) described WTAP in their literature as a protective factor, and the opposite was true for Tang et al. (2018)

Advances in science and technology in recent years have deepened our horizon of the immune system and its response to tumor cells. The tumor immunotherapy is to employ passive or active immunity against malignant tumors by using the immune system to precisely target tumors (Xiong and Wang, 2022). We also notice the term “TME,” which infers to the tumor microenvironment (TME). Apart from stromal cells, fibroblasts, and endothelial cells, innate and adaptive immune cells are also included in TME. Unlike the conventional concept about immune cells in our mind, more evidence suggests that the innate immune cells (macrophages, neutrophils, dendritic cells, innate lymphoid cells, myeloid-derived suppressor cells, and natural killer cells) as well as adaptive immune cells (T cells and B cells) corelate with tumor progression when present in the TME (Hinshaw and Shevde, 2019). To be concrete, tumor-associated macrophages (TAMs) are widely infiltrating immune cells in the TME which is in close relationship with adverse outcomes of cancers in most circumstances. The biological effects of TAMs are various and abundant, contributing to angiogenesis, tumor invasion and metastasis, and an immunosuppressive microenvironment (Cheng et al., 2021). Given what is listed above, immunotherapies are emerging continuously. Take CAR-T cells for example, T cells modified to express chimeric antigen receptors (CARs) with tumor specificity have been reported remarkable success in helping patients with hematologic malignancies and revitalized the field of adoptive cell therapy. It is a remarkable new achievement in cancer therapy. Treatment with CAR-T cells has acquired positive clinical responses in certain subsets of B cell leukemia or lymphoma (Hong et al., 2020; Sterner and Sterner, 2021). In addition, there are also other ones like cytotoxic T lymphocyte associated antigen 4 (CTLA4), programmed death-1 (PD-1), and programmed death ligand-1 (PD-L1). Satisfactory results have been obtained in the treatment of malignant melanoma and non-small-cell lung carcinoma (Topalian et al., 2015).

Through this work, we use different databases, including PrognoScan, GEPIA, Timer, and Kaplan-Meier Plotter to reflect and analyze the WTAP-related prognosis of WTAP in pan-cancer. Moreover, we explain the relationship between WTAP expression level and immune cell infiltration level with TIMER and GEPIA online tools. This study indicates WTAP expression level is correlated with the prognosis of various cancer which is possibly resulted from infiltration of immune cells.

## Materials and methods

### Survival analysis in PrognoScan, GEPIA, and Kaplan-Meier Plotter

PrognoScan (http://dna00.bio. kyutech.ac.jp/PrognoScan/index.html), Kaplan-Meier Plotter (https://kmplot.com/analysis/), and GEPIA (http://gepia.cancer-pku.cn/) were used to analysis the relationship of WTAP expression and prognosis (Mizuno et al., 2009; Tang et al., 2017; Nagy et al., 2018). To be more specific, all available microarray datasets in PrognoScan were retrieved under the gene symbol WTAP, to determine the possible correlation between survival indicator overall survival (OS), disease-free survival (DFS) and WTAP expression level. The threshold was set as a Cox *p*-value < 0.05, and data collected from PrognoScan was analyzed and visualized with the “ggplot2” package in R software (version 3.25.0, www.r-project.org). GEPIA is an interactive online server with RNA sequencing data of tumor sample from TCGA and normal sample from both The Cancer Genome Atlas (TCGA) and GTEx. The impacts of WTAP expression on OS and DFS in all available cancer types (total number = 34) were then studied. Kaplan-Meier Plotter is a strong online tool used for validation of survival biomarkers in 21 tumor types. Overall survival (OS) and relapse-free survival (RFS) in bladder carcinoma (BC), BRCA, cervical squamous cell carcinoma (CSCC), esophageal squamous cell carcinoma (ESCC), kidney renal clear cell carcinoma (KIRC), LIHC, ovarian carcinoma (OVC), and rectum adenocarcinoma (READ) were plotted depending on its relationship with WTAP expression. Hazard ratios (HRs) with 95% confidence intervals (CI) and log-rank *p*-values were calculated.

### Correlations between Wilms tumor 1-associated protein expression and immune cells in TIMER and GEPIA

Using TIMER (http://cistrome.org/TIMER/) and GEPIA databases, the association between WTAP expression and immune infiltration was studied (Li et al., 2016; Tang et al., 2017). TIMER is a systematic resource for exploration of association between immune infiltration and genomic/clinicopathological characteristics among diverse cancer types. The abundance of tumor-infiltrating immune cells or specific cell marker are estimated by a statistical deconvolution method from gene expression profiles in TCGA tumor samples. We analyzed WTAP expression with six common types of immune infiltrating cells, including B cells, CD4^+^ T cells, CD8^+^ T cells, neutrophils, macrophages, and dendritic cells. Tumor purity was also considered as a variable of WTAP expression.

To figure out the specific relationship of WTAP expression with immune cell subgroups, immune cell markers were identified from the online tools of R&D Systems (https://www.rndsystems.com/cn/resources/cell-markers/immune-cells). Markers of B cells, CD8^+^ T cells, follicular helper T cells (Tfh), T-helper 1 (Th1) cells, T-helper 2 (Th2) cells, T-helper 9 (Th9) cells, T-helper 17 (Th17) cells, T-helper 22 (Th22) cells, Tregs, exhausted T cells, M1 macrophages, M2 macrophages, tumor-associated macrophages, monocytes, natural killer (NK) cells, neutrophils, and dendritic cells were selected and examined with WTAP expression. Log2 TPM was used to adjust marker genes expression level. WTAP was plotted on the *x*-axis, while marker genes were plotted on the *y*-axis. Relationship of WTAP and each immune gene marker was illustrated in the form of scatterplot. Gene expression correlation analysis was also performed similarly in GEPIA according to expression dataset of interest. The non-log scale is used for calculation and the log-scale axis is used for visualization. The correlation coefficient was determined by spearman method. Correlation between WTAP and immune markers was then plotted.

### Statistical analysis

The survival curve was estimated and fitted by Kaplan-Meier method. To compare the difference between survival curves of high/low expression groups, we calculated hazard ratio and logrank *p*-value in Kaplan-Meier Plotter and GEPIA. A univariate Cox regression model was applied to calculate the hazard ratio and Cox *p* value in PrognoScan. The significance of gene correlation analysis was evaluated with Spearman’s correlation. *p* < 0.05 was considered statistically significant.

## Results

### mRNA expression level of Wilms tumor 1-associated protein in pan-cancer

**FIGURE 1:**
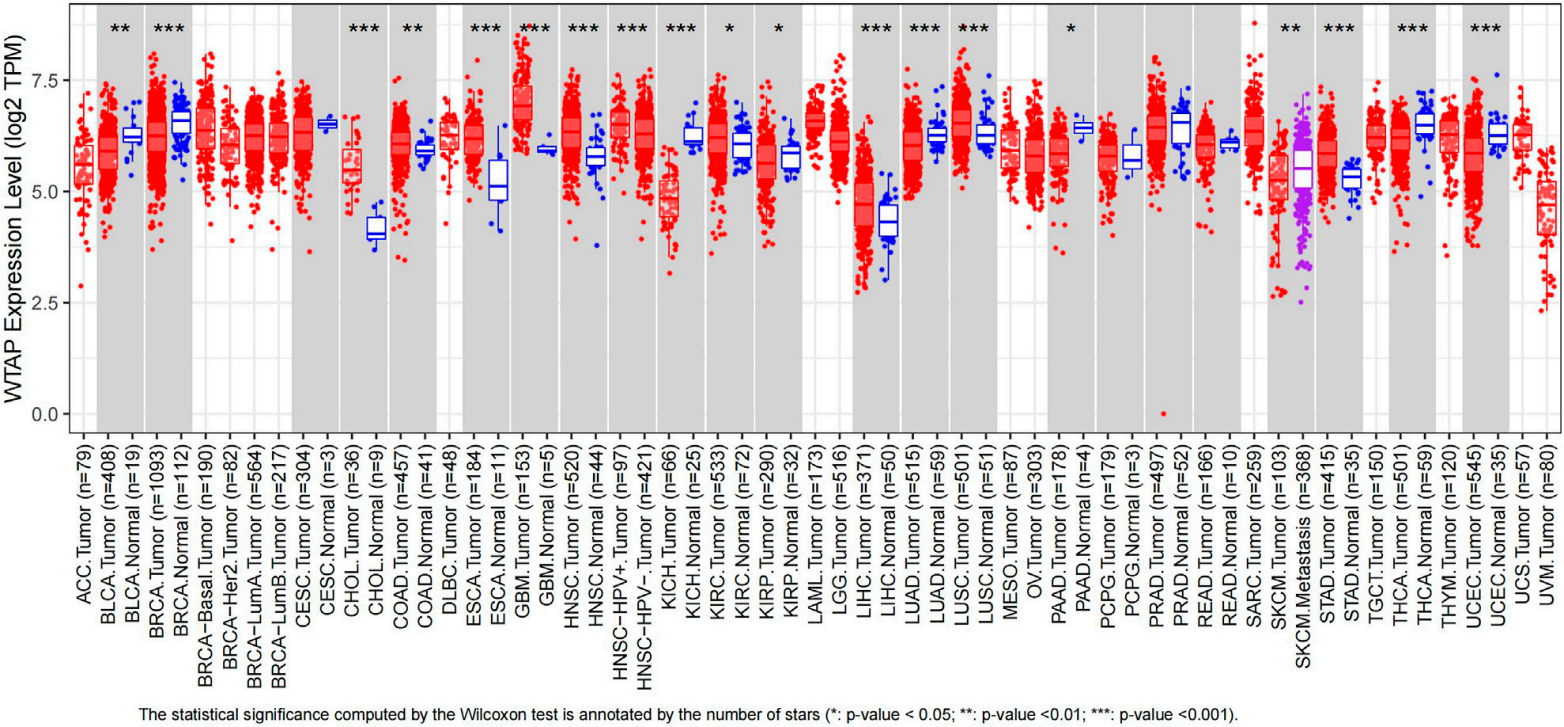
Human WTAP expression levels in different types of cancer from TCGA data in TIMER. Gray rectangles represent cancer types. Red patterns stand for tumor samples while blue patterns stand for normal samples. Purple pattern represents metastasis samples. Some cancer types are short of normal samples and corresponding *p* value cannot be calculated. *p < 0.05, **p < 0.01, ***p < 0.001. (ACC, adrenocortical carcinoma; BLCA, bladder urothelial carcinoma; BRCA, breast invasive carcinoma; CESC, cervical squamous cell carcinoma and endocervical adenocarcinoma; CHOL, cholangiocarcinoma; COAD, colon adenocarcinoma; DLBC, lymphoid neoplasm diffuse large B-cell lymphoma; ESCA, esophageal carcinoma; GBM, glioblastoma multiforme; HNSC, head and neck squamous cell carcinoma; KICH, kidney chromophobe; KIRC, kidney renal clear cell carcinoma; KIRP, kidney renal papillary cell carcinoma; LAML, acute myeloid leukemia; LGG, brain lower grade glioma; LIHC, liver hepatocellular carcinoma; LUAD, lung adenocarcinoma; LUSC, lung squamous cell carcinoma; MESO, mesothelioma; OV, ovarian serous cystadenocarcinoma; PAAD, pancreatic adenocarcinoma; PCPG, pheochromocytoma and paraganglioma; PRAD, prostate adenocarcinoma; READ, rectum adenocarcinoma; SARC, sarcoma; SKCM, skin cutaneous melanoma; STAD, stomach adenocarcinoma; TGCT, testicular germ cell tumors; THCA, thyroid carcinoma; THYM, thymoma; UCEC, uterine corpus endometrial carcinoma; UCS, uterine carcinosarcoma; UVM, uveal melanoma).

Using TIMER, we evaluated the expression levels of WTAP from its RNA sequencing data in TCGA. The results were shown in Figure 1. The graph showed that WTAP expression level was significantly higher in CHOL (cholangiocarcinoma), COAD (colon adenocarcinoma), ESCA, GBM (glioblastoma multiforme), HNSC-HPV (head and neck squamous cell carcinoma), KIRC, LIHC, LUSC (lung squamous cell carcinoma), STAD (stomach adenocarcinoma) than in normal tissue, while significantly lower in BLCA, BRCA, KICH (kidney chromophobe), KIRP (kidney renal papillary cell carcinoma), PAAD (pancreatic adenocarcinoma), SKCM (skin cutaneous melanoma) compared with their adjacent normal tissues. Due to the lack of normal samples in TCGA, we integrated the normal samples from the GTEx database to increase the credibility of significance from GEPIA. Diffuse large B-cell lymphoma (DLBC), GBM, Thymoma (THYM) showed significantly higher expression of WTAP compared to normal tissues, while KICH showed the opposite trend (Supplementary Figure S1).

### Multifaceted prognostic value of Wilms tumor 1-associated protein in cancers

Our previous study showed high expression level of WTAP was associated with increased incidence of liver cancer and adverse prognosis. In this study, the prognostic value of WTAP for pan-cancer was investigated. We explored the relationships between WTAP expression level and the prognosis of cancer of interest.

**FIGURE 2:**
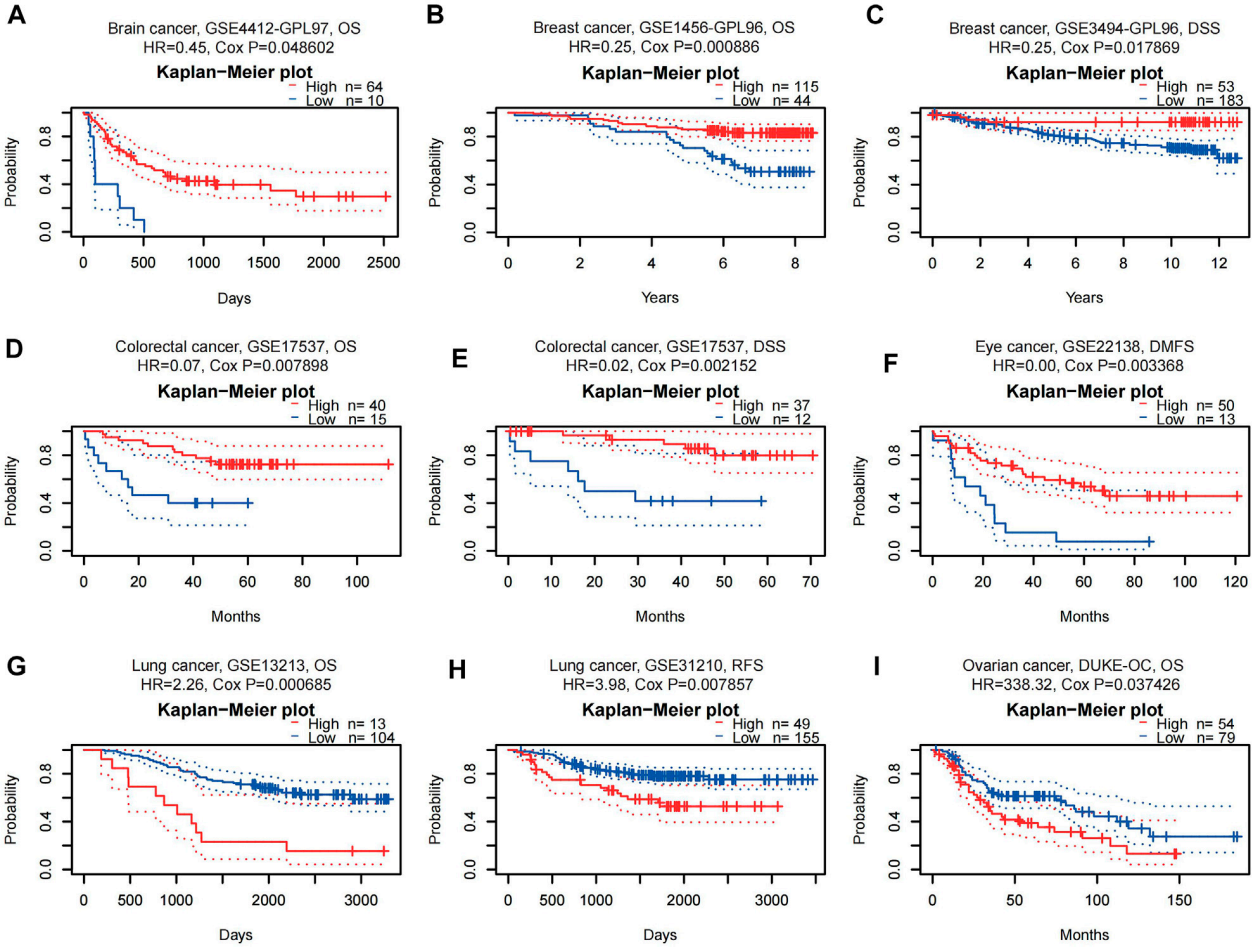
Kaplan-Meier survival curves comparing high and low expression of WTAP in different cancer types in PrognoScan. **(A)** OS (*n* = 74, cutpoint = 0.14) in brain cancer cohort CSE4412-GPL97. **(B,C)** OS (*n* = 159, cutpoint = 0.43) and DSS (*n* = 236, cutpoint = 0.77) in breast cancer cohort GSE1456-GPL96 and GSE3494-GPL96. **(D,E)** OS (*n* = 55, cutpoint = 0.25) and DSS (*n* = 49, cutpoint = 0.24) in colorectal cancer cohort GSE17537. **(F)** DMFS (*n* = 63, cutpoint = 0.21) in eye cancer cohort GSE22138. **(G,H)** OS (*n* = 117, cutpoint = 0.89) and RFS (*n* = 204, cutpoint = 0.76) in lung cancer cohort GSE13213 and GSE31210. **(I)** OS (*n* = 133, cutpoint = 0.58) in ovarian cancer cohort DUKE-OC. OS, overall survival; DSS, disease-specific survival; DMFS, distant metastasis-free survival; RFS, relapse-free survival.

WTAP expression significantly correlated with a total of 11 prognostic values in six cancer types, including brain, breast, colorectal, eye, lung and ovarian cancers (Figure 2), nine of them were listed below. Among them, WTAP played a protective role in four cancer types, including brain (OS: total number = 74, HR = 0.45, Cox *p* = 0.048602), breast [DSS(disease-specific survival): total number = 236, HR = 0.25, Cox *p* = 0.017869; OS: total number = 159, HR = 0.25, Cox *p* = 0.000886], colorectal [DSS: total number = 49, HR = 0.02, Cox *p* = 0.002152; OS: total number = 55, HR = 0.07, Cox *p* = 0.007898], and eye cancers [DMFS(distant metastasis-free survival): total number = 63, HR = 0.00, Cox *p* = 0.003368]. Meanwhile, WTAP had a detrimental role in other two cancer types, including lung cancers [OS: total number = 117, HR = 2.26, Cox *p* = 0.000685; RFS (relapse-free survival), total number = 204, HR = 3.98, Cox *p* = 0.007857], and ovarian cancers (OS: total number = 133, HR = 338.32, Cox *p* = 0.037426).

**FIGURE 3:**
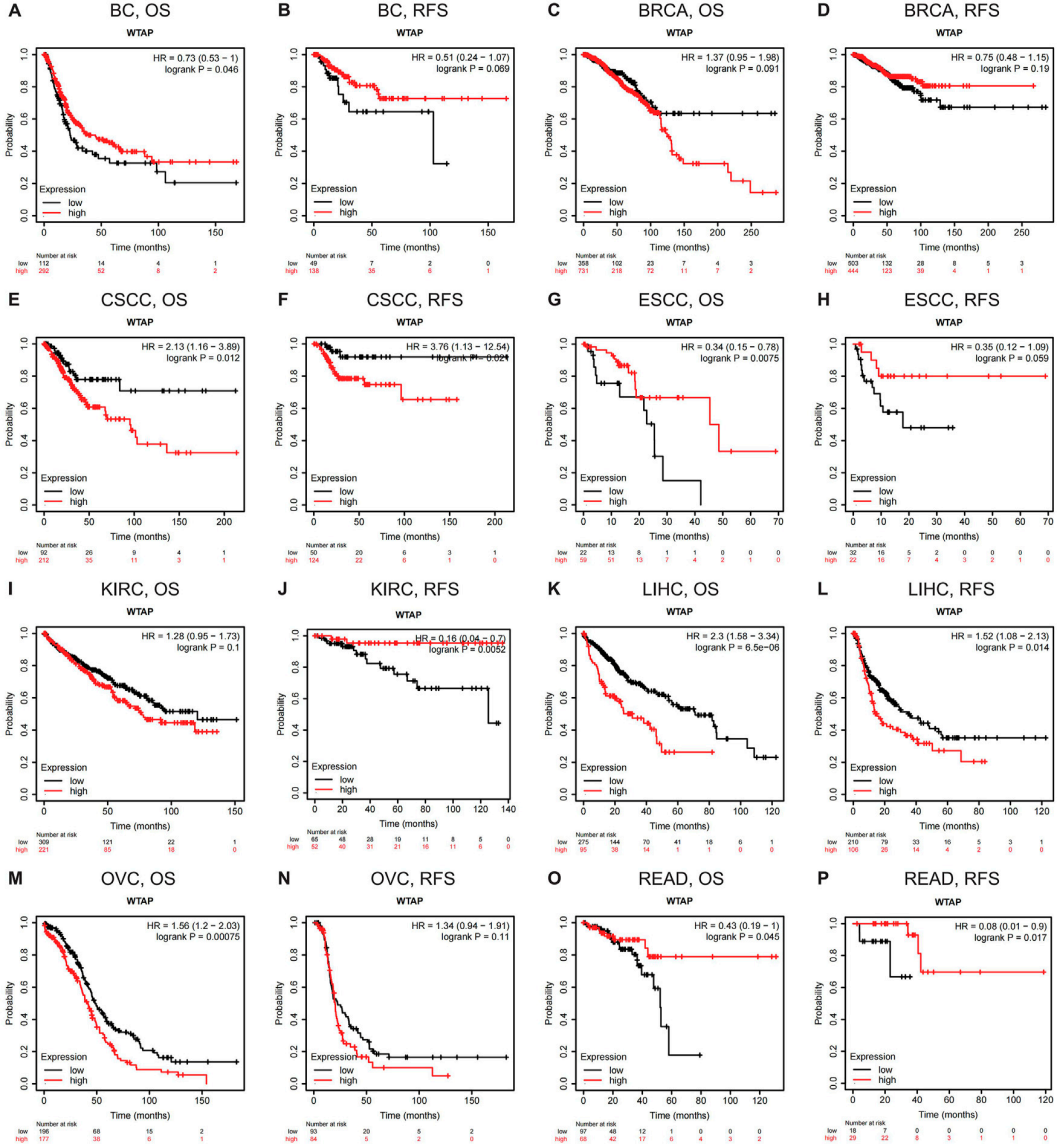
Kaplan-Meier survival curves comparing the high and low expression of WTAP in different types of cancer in Kaplan-Meier Plotter. OS and RFS of **(A,B)** bladder carcinoma (BC) **(C,D)** breast cancer (BRCA) **(E,F)** cervical squamous cell carcinoma (CSCC) **(G,H)** esophageal squamous cell carcinoma (ESCC) **(I,J)** kidney renal clear cell carcinoma (KIRC) **(K,L)** liver hepatocellular carcinoma (LIHC) **(M,N)** ovarian cancer (OVC), and **(O,P)** rectum adenocarcinoma (READ). Red curve represents patients with high expression of WTAP. OS, overall survival; RFS, relapse-free survival.

The databases of PrognoScan are based on gene expression omnibus (GEO). For a more complete and rigorous analysis, Kaplan-Meier Plotter, another database based on Affymetrix microarray information from TCGA, is used to evaluate WTAP-related survival (OS and RFS) (Figure 3). For LIHC, WTAP significantly worsens its overall survival and relapse-free survival (LIHC: OS, HR = 2.3, 95% CI from 1.58 to 3.34, logrank *p* = 6.5e-06; RFS, HR = 1.52, 95% CI from 1.08 to 2.13, logrank *p* = 0.014). For READ, WTAP has a protective effect on both overall survival and relapse-free survival (READ: OS, HR = 0.43, 95% CI from 0.19 to 1, logrank *p* = 0.045; RFS, HR = 0.08, 95% CI from 0.01 to 0.9, logrank *p* = 0.017). For both CSCC and KIRC, WTAP significantly influences their relapse-free survival, protective on KIRC while detrimental on CSCC (KIRC: RFS, HR = 0.16, 95% CI from 0.04 to 0.7, logrank *p* = 0.0052; CSCC: RFS, HR = 3.76, 95% CI from 1.13 to 12.54, logrank *p* = 0.024). For ESCC and BC, with higher WTAP expression, the overall survival gets better (ESCC: OS, HR = 0.34, 95% CI from 0.15 to 0.78, logrank *p* = 0.0075; BC: OS, HR = 0.73, 95% CI from 0.53 to 1, logrank *p* = 0.046). For ovarian cancer (OVC), WTAP worsens its overall survival (OS, HR = 1.56, 95% CI from 1.2 to 2.03, logrank *p* = 0.00075). Moreover, the findings of BRCA are partially different from those in PrognoScan. WTAP is found to have a protective role in breast cancer in PrognoScan while no significant relationship is discovered in Kaplan-Meier Plotter. The selection of different data resources and cohorts for analysis may account for the instability.

### Expression of Wilms tumor 1-associated protein among different stratified liver hepatocellular carcinoma population

Seeing the relevance between WTAP expression and its prognostic value in LIHC, we attempted to explore the potential relevance and mechanisms of WTAP expression in LIHC. Kaplan-Meier Plotter is a powerful database, which provides us clinical and pathological data in LIHC patients. By integrating the data, we further explored the relationship of WTAP expression to several clinical features in LIHC patients.

**FIGURE 4:**
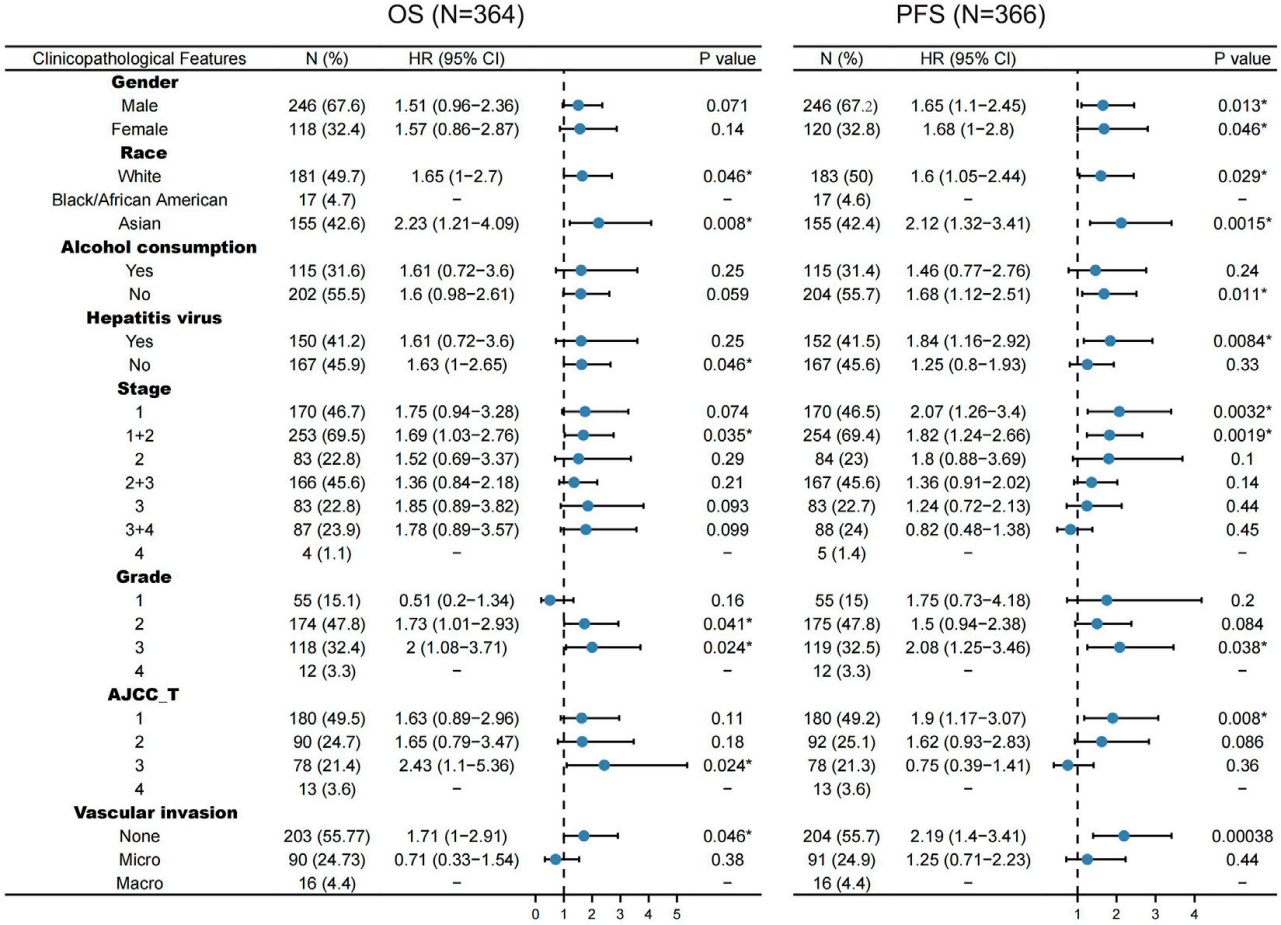
Correlation of WTAP mRNA expression with OS (*n* = 364) and PFS (*n* = 366) in liver hepatocellular carcinoma with various clinicopathological features. Blue dots stand for hazard ratio. Short bars appear due to small sample capacity for parameters, so that hazard ratio and corresponding *p* value cannot be calculated. OS, overall survival; PFS, progression free survival. *p < 0.05.

For OS, WTAP exert a harmful effect on LIHC patient who possess the following characteristics: White (N = 181, HR = 1.65, 95% CI from 1 to 2.7, *p* = 0.046), Asian (N = 155, HR = 2.23, 95% CI from 1.21 to 4.09, *p* = 0.008), no hepatitis virus infection (N = 167, HR = 1.63, 95% CI from 1 to 2.65, *p* = 0.046), LIHC patients at 1 + 2 stage (N = 253, HR = 1.69, 95% CI from 1.03 to 2.76, *p* = 0.035), Grade 2 (N = 174, HR = 1.73, 95% CI from 1.01 to 2.93, *p* = 0.041), Grade 3 (N = 118, HR = 2, 95% CI from 1.08 to 3.71, *p* = 0.024), American Joint Committee on Cancer (AJCC) T3 stage (N = 78, HR = 2.43, 95% CI from 1.1 to 5.36, *p* = 0.024), and none vascular invasion (N = 203, HR = 1.71, 95% CI from 1 to 2.91, *p* = 0.046) (Figure 4).

For PFS, WTAP also play a detrimental role in the following LIHC patients: male (N = 246, HR = 1.65, 95% CI from 1.1 to 2.45, *p* = 0.013), female (N = 120, HR = 1.68, 95% CI from 1 to 2.8, *p* = 0.046), White (N = 183, HR = 1.6, 95% CI from 1.05 to 2.44, *p* = 0.029), Asian (N = 155, HR = 2.12, 95% CI from 1.32 to 3.41, *p* = 0.0015), no alcohol consumption (N = 204, HR = 1.68, 95% CI from 1.12 to 2.51, *p* = 0.011), hepatitis virus infection (N = 152, HR = 1.84, 95% CI from 1.16 to 2.92, *p* = 0.0084), LIHC patients at 1 stage (N = 170, HR = 2.07, 95% CI from 1.26 to 3.4, *p* = 0.032), LIHC patients at 1 + 2 stage (N = 254, HR = 1.82, 95% CI from 1.24 to 2.66, *p* = 0.0019), Grade 3 (N = 119, HR = 2.08, 95% CI from 1.25 to 3.46, *p* = 0.038), AJCC T1 stage (N = 180, HR = 1.9, 95% CI from 1.17 to 3.07, *p* = 0.008) (Figure 4).

### Opposite results for liver hepatocellular carcinoma and esophageal carcinoma in Wilms tumor 1-associated protein induced immune cell infiltration

The above finding supports the widely prognostic role of WTAP in pan-cancer, and we suppose that immune cell infiltration in TME may contribute to the WTAP-induced survivor indicator change.

Therefore, it would be important to explore the association between immune cell infiltration in the tumor microenvironment and WTAP expression.

**FIGURE 5:**
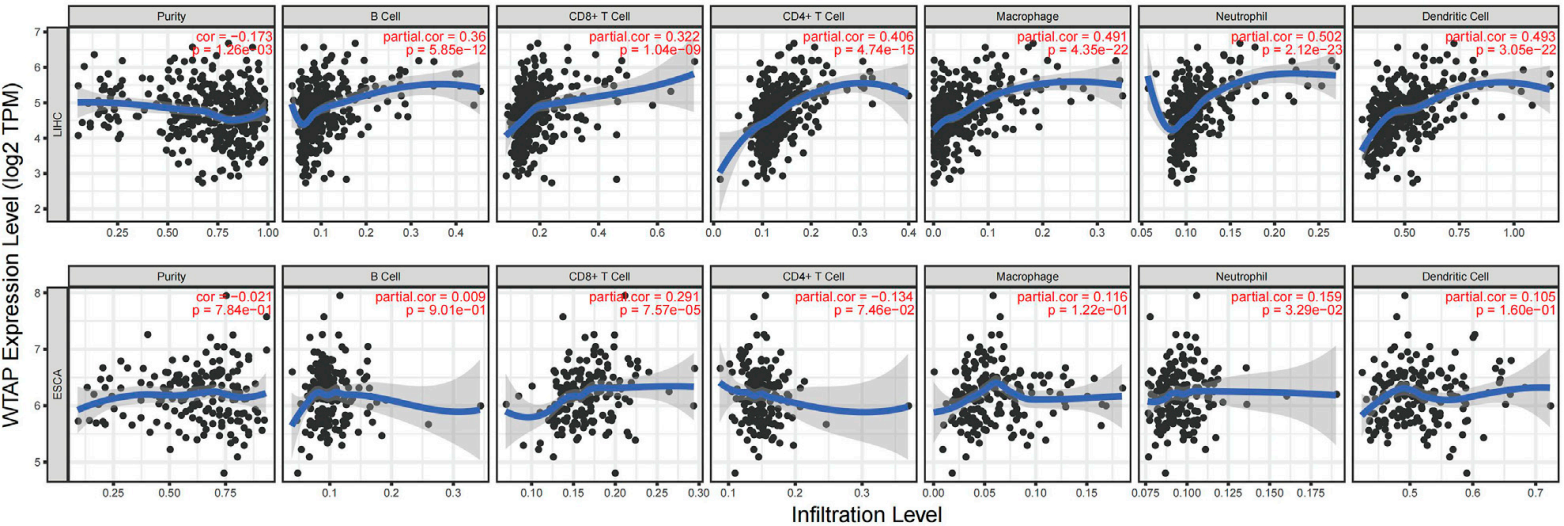
Correlation of WTAP expression with immune infiltration level in LIHC and ESCA. Top row: WTAP expression has noteworthy negative relation with tumor purity and significant positive correlation with infiltrating levels of B cell, CD8^+^ T cell, CD4^+^ T cell, macrophage, neutrophil, and dendritic cell in LIHC. Bottom row: In ESCA, WTAP expression has significant positive correlation with infiltrating levels of CD8^+^ T cell and neutrophil, but no obvious relationship with tumor purity and infiltrating levels of B cell, CD4^+^ T cell, macrophage, and dendritic cell. LIHC, liver hepatocellular carcinoma; ESCA, esophageal carcinoma. *p* < 0.05 is considered as significant.

By calculating WTAP expression coefficients and immune infiltration levels in LIHC and ESCA, we attempted to figure out whether WTAP expression correlates with immune cell infiltration levels in tumors. As the results shows, WTAP expression significantly correlated with tumor purity in LIHC but not ESCA. Additionally, WTAP expression also significantly assocaited with the infiltration levels of B cells, CD8^+^ T cells, CD4^+^ T cells, macrophages, neutrophils and dendritic cells in LIHC, while in ESCA only CD8^+^ T cells and neutrophils show significant correlation with WTAP level (Figure 5).

TIMER and GEPIA share most of the homologous data from TCGA. Based on the above findings in GEPIA, when WTAP expression was at a high level, we picked LIHC to represent cancers with poor survival and ESCC to represent cancers with good survival. Due to the limit of samples, we analyzed ESCA instead, in which 90% of cases are ESCC approximately (Arnold et al., 2015). For LIHC, WTAP expression shows strongly positive correlations with the infiltration level of B cells (R = 0.36, *p* = 5.85E–12), CD8^+^ T cells (R = 0.322, *p* = 1.04E–09), CD4^+^ T cells (R = 0.406, *p* = 4.74E−15), macrophages (R = 0.491, *p* = 4.35E–22), neutrophils (R = 0.502, *p* = 2.12E–23), and dendritic cells (R = 0.493, *p* = 3.05E−33) (Figure 5). Similarly, for ESCA, WTAP expression also shows positive correlations with the infiltration level of CD8^+^ T cells (R = 0.291, *p* = 7.57E-05) and neutrophils (R = 0.159, *p* = 3.29E–02). Nevertheless, compared to LIHC, we can also see that the infiltration level of B cells, CD4^+^ T cells, macrophages and dendritic cells show no significance in ESCA (Figure 5). Moreover, in LIHC, WTAP expression shows a negative correlation with tumor purity which indicates the multifaceted function of WTAP in tumor development. Thus, immune cells gene marker expression adjusted for tumor purity is listed (Table1; Figure 5). These findings indicate that WTAP can influence immune infiltration in the tumor microenvironment in cancers such as LIHC, especially the infiltration level of CD4^+^ T cells, macrophages and dendritic cells, which possibly affect patient survival. The detailed mechanisms of how WTAP-related immune infiltration put a hazardous effect on tumor prognosis remain to be studied.

**TABLE 1:** Correlations between WTAP and gene markers of immune cells in LIHC and ESCA.

Cell type	Gene marker	LIHC				ESCA			
		None		Purity		None		Purity	
		Cor	*P*	Cor	*P*	Cor	*P*	Cor	*P*
B cell	CD19	0.303	***	0.293	***	−0.004	0.961	0.007	0.930
	MS4A1	0.255	***	0.243	***	−0.015	0.843	0.001	0.989
	CD38	0.396	***	0.402	***	0.166	0.024	0.171	0.021
CD8^+^ T cell	CD8A	0.327	***	0.322	***	0.161	0.028	0.190	0.010
	CD8B	0.238	***	0.246	***	0.080	0.278	0.107	0.152
Tfh	CXCR5	0.340	***	0.339	***	0.001	0.993	0.018	0.810
	ICOS	0.396	***	0.405	***	0.320	***	0.339	***
	BCL6	0.411	***	0.413	***	0.240	*	0.228	*
Th1	IL12RB2	0.324	***	0.314	***	0.299	***	0.315	***
	IL27RA	0.483	***	0.481	***	0.352	***	0.341	***
	TBX21	0.279	***	0.293	***	0.136	0.065	0.160	0.031
Th2	CCR3	0.336	***	0.334	***	−0.045	0.546	−0.047	0.527
	STAT6	0.367	***	0.358	***	0.146	0.047	0.149	0.045
	GATA3	0.382	***	0.398	***	0.112	0.130	0.107	0.151
Th9	TGFBR2	0.407	***	0.419	***	0.028	0.707	0.024	0.750
	IRF4	0.413	***	0.418	***	0.127	0.086	0.140	0.060
	SPI1	0.471	***	0.471	***	0.165	0.025	0.171	0.022
Th17	IL21R	0.464	***	0.469	***	0.187	0.011	0.196	*
	IL23R	0.319	***	0.321	***	0.087	0.241	0.093	0.212
	STAT3	0.547	***	0.554	***	0.356	***	0.346	***
Th22	CCR10	0.358	***	0.367	***	−0.023	0.752	−0.013	0.866
	AHR	0.299	***	0.303	***	0.264	**	0.256	**
Treg	FOXP3	0.275	***	0.280	***	0.266	**	0.286	***
	CCR8	0.494	***	0.505	***	0.244	**	0.258	**
	IL2RA	0.499	***	0.495	***	0.330	***	0.342	***
T cell exhaustion	PDCD1	0.338	***	0.328	***	0.162	0.028	0.186	0.012
	CTLA4	0.362	***	0.367	***	0.313	***	0.335	***
Macrophage	CD68	0.376	***	0.374	***	0.083	0.258	0.090	0.230
	ITGAM	0.441	***	0.451	***	0.216	*	0.213	*
M1	NOS2	0.167	*	0.169	*	−0.100	0.177	−0.125	0.095
	ROS1	0.209	***	0.206	**	0.236	*	0.251	**
M2	ARG1	−0.022	0.675	−0.026	0.624	0.008	0.918	0.029	0.698
	MRC1	0.221	***	0.220	***	0.104	0.159	0.121	0.106
TAM	HLA-G	0.228	***	0.223	***	−0.064	0.385	−0.059	0.431
	CD80	0.537	***	0.539	***	0.285	***	0.291	***
	CD86	0.486	***	0.487	***	0.292	***	0.301	***
Monocyte	CD14	−0.201	***	−0.180	**	0.126	0.088	0.123	0.099
	FCGR3A	0.456	***	0.465	***	0.235	*	0.253	**
NK	XCL1	0.214	***	0.230	***	0.194	*	0.194	*
	KIR3DL1	0.190	**	0.199	**	0.071	0.340	0.082	0.273
	CD7	0.227	***	0.225	***	0.169	0.022	0.186	0.012
Neutrophil	FUT4	0.542	***	0.541	***	−0.027	0.710	−0.016	0.835
	MPO	0.272	***	0.266	***	−0.005	0.942	0.007	0.921
DC	CD1C	0.292	***	0.286	***	0.073	0.326	0.101	0.177
	THBD	0.319	***	0.306	***	0.250	**	0.259	**

LIHC, liver hepatocellular carcinoma; ESCA, esophageal carcinoma; Tfh, follicular helper T cell; Th, T helper cell; Treg, regulatory T cell; M1/M2, macrophages; TAM, tumor-associated macrophage; NK, natural killer cell; DC, dendritic cell; None, correlation without adjustment; Purity, correlation adjusted for tumor purity; Cor, R value of Spearman’s correlation. *p < 0.01; **p < 0.001; ***p < 0.0001.

### Relationships between Wilms tumor 1-associated protein expression and immune markers

**FIGURE 6:**
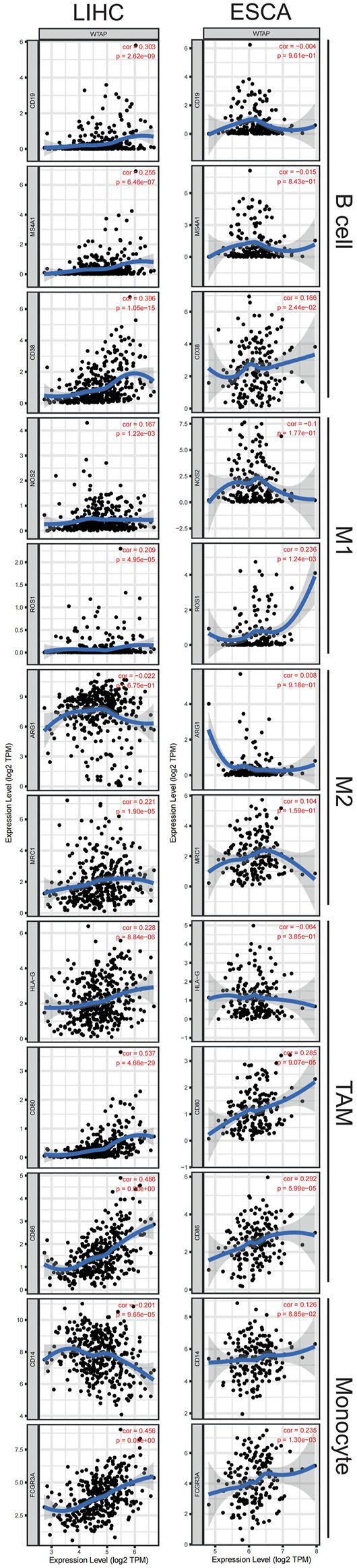
Correlation of WTAP expression with immune cell specific gene markers in LIHC and ESCA. Immune cells, including B cells, M1/M2 macrophages, tumor-associated macrophages (TAM), monocytes are characterized respectively in LIHC and ESCA. Markers include CD19, MS4A1, and CD38 of B cell, NOS2 and ROS1 of M1 macrophage, ARG1 and MRC1 of M2 macrophage, HLA-G, CD80, and CD86 of TAM, and CD14 and FCGR3Aof monocyte. LIHC, liver hepatocellular carcinoma; ESCA, esophageal carcinoma; TAM, tumor-associated-macrophages. *p* < 0.05 is considered as significant.

After a glimpse into the relationships between WTAP and immune infiltration, we further exploited TIMER and GEPIA for association between WTAP expression level and immune cell specific gene markers. Immune cells, including B cells, CD8^+^ T cells, M1/M2 macrophages, TAM, monocytes, NK, neutrophils, and DCs are characterized respectively in LIHC and ESCA. Subgroups of T cells were examined as well, including Tfh, Th1, Th2, Th9, Th17, Th22, Treg, and exhausted T cells (Table 1; Figure 6). 44 out of 45 immune cell markers in LIHC had significant correlation with WTAP expression in LIHC while only 18 out of 45 immune cell markers showed significance in ESCA after adjustment for tumor purity (Table 1). As demonstrated by Figure 5, in LIHC, B cells, CD4^+^ T cells, macrophages, and dendritic cells were strongly correlated with WTAP content, which were not significant in ESCA. The similar discrepancy between immune cells infiltration in TME of LIHC and ESCA was also demonstrated in Table 1, different correlations between WTAP expression and B cells/CD4+ T cells/macrophages/dendritic cells markers were listed. Therefore, to comprehensively explain the relationships of WTAP and immune cells infiltration, we further analyzed the correlations of WTAP expression and gene markers in normal and tumor tissues of LIHC and ESCA in GEPIA. The results suggested that WTAP correlates with T reg infiltration in LIHC and ESCA. WTAP expression in normal tissue could affect immune infiltration as well (Table 2).

**TABLE 2:** Correlations between WTAP and genes markers of normal and tumor tissues of LIHC and ESCA.

Gene marker	LIHC				ESCA			
	Tumor		Normal		Tumor		Normal	
	R	P	R	P	R	P	R	P
CD19	0.300	***	0.220	0.120	−0.057	0.450	0.250	0.400
CD20 (MS4A1)	0.260	***	0.390	**	−0.070	0.350	−0.210	0.490
CD38	0.400	***	0.520	***	0.120	0.120	0.470	0.110
CD8A	0.320	***	0.460	***	0.092	0.220	0.200	0.520
CD8B	0.240	***	0.310	*	0.009	0.900	0.055	0.860
CXCR5	0.140	**	0.670	0.061	0.045	0.550	0.770	**
ICOS	0.380	***	0.460	***	0.270	***	0.460	0.110
BCL6	0.410	***	0.640	***	0.260	***	0.720	**
IL12RB2	0.360	***	0.380	**	0.310	***	0.610	*
IL27RA	0.470	***	0.720	***	0.320	***	0.009	0.710
TBX21	0.290	***	0.440	**	0.066	0.380	0.330	0.270
CCR3	0.340	***	0.130	0.350	−0.037	0.620	0.660	*
STAT6	0.410	***	0.610	***	0.180	*	0.900	***
GATA3	0.400	***	0.590	***	0.047	0.530	0.460	0.120
TGFBR2	0.430	***	0.800	***	0.039	0.600	0.740	**
IRF4	0.400	***	0.880	*	0.054	0.470	−0.016	0.960
SPI1	0.470	***	0.610	***	0.110	0.150	0.790	**
IL21R	0.480	***	0.460	***	0.100	0.160	0.500	0.079
IL23R	0.330	***	0.340	*	0.060	0.420	0.540	0.058
STAT3	0.570	***	0.620	***	0.390	***	0.910	***
CCR10	0.350	***	0.310	*	−0.036	0.630	0.240	0.430
AHR	0.310	***	0.470	***	0.310	***	0.870	***
FOXP3	0.190	***	−0.045	0.760	0.210	**	0.390	0.190
CCR8	0.480	***	0.220	0.120	0.200	**	0.160	0.600
IL2RA	0.490	***	0.450	**	0.270	***	0.780	**
PDCD1	0.330	***	0.350	*	0.093	0.210	0.430	0.140
CTLA4	0.370	***	0.360	**	0.240	**	0.310	0.310
CD68	0.400	***	0.650	***	0.009	0.210	0.850	***
ITGAM	0.470	***	0.430	**	0.200	**	0.870	***
NOS2	0.210	***	0.230	0.110	−0.120	0.120	0.870	***
ROS	0.210	***	0.130	0.390	0.260	***	−0.180	0.550
ARG1	0.016	0.760	0.240	0.091	0.039	0.600	0.640	*
MRC1	0.270	***	0.430	**	0.088	0.240	0.270	0.370
HLA-G	0.280	***	0.290	*	0.003	0.970	0.580	*
CD80	0.510	***	0.640	***	0.260	***	0.590	*
CD86	0.500	***	0.660	***	0.250	***	0.580	*
CD14	−0.16	**	0.300	*	0.096	0.200	0.780	**
CD16 (FCGR3A)	0.460	***	0.450	**	0.210	**	0.720	**
XCL1	0.220	***	0.430	**	0.180	*	0.350	0.250
KIR3DL1	0.170	**	0.350	*	0.030	0.680	0.600	*
CD7	0.210	***	0.330	*	0.100	0.160	0.340	0.250
FUT4	0.530	***	0.690	***	−0.051	0.490	0.610	*
MPO	0.310	***	0.320	*	−0.018	0.810	0.400	0.180
CD1C	0.300	***	0.160	0.250	0.028	0.710	0.470	0.110
THBD	0.380	***	0.670	***	0.320	***	0.220	0.470

LIHC, liver hepatocellular carcinoma; ESCA, esophageal carcinoma; Tfh, follicular helper T cell; Th, T helper cell; Treg, regulatory T cell; TAM, tumor-associated macrophage; NK, natural killer cell; Tumor, correlation analysis in tumor tissue of TCGA; normal, correlation analysis in normal tissue of TCGA. *p < 0.01; **p < 0.001; ***p < 0.0001.

Besides, WTAP expression in LIHC and ESCA also correlated differently with CD8^+^ T cell, Tfh, Th2, Th9, Treg, exhausted T cells infiltration (Table 1) Cell specific markers of Th1 cells, Th17 cells, Th22 cells, macrophage, neutrophils, M1, M2, TAM, monocytes, NK cells and dendritic cells were differently correlated to WTAP expression to some extent between LIHC and ESCA. It is worth noting that WTAP expression showed a significant link with programmed cell death protein 1 (PDCD1), marker of T cell exhaustion, in LIHC which can potentially function as a therapeutic target while no such correlation was seen in ESCA (James et al., 2005). These results were consistent with our hypothesis that influence on prognosis led by WTAP could potentially result from WTAP dependent immune cell infiltration level.

## Discussion

Methylation of N6 adenosine (m6A) in RNA is a methylation modification that occurs on the sixth N atom of RNA adenine, which is highly prevalent and conserved, playing a critical role in post-transcriptional regulation (Wang and Zhao, 2016; Duan et al., 2019). As the most important RNA epigenetic regulation in eukaryotic cells, the m6A modification affects RNA metabolism and plays an essential role in physiological as well as pathological conditions (Dai et al., 2018; Jiang et al., 2021). Existing studies suggest that m6A mRNA not only acts on tumor cells, including on proliferation, stemness, invasion capability, but also interacts with the TME (Li et al., 2021). Nowadays, m6A methylation is one of the hot topics in tumor immunity research.

WTAP is a conserved nuclear protein, part of the m6A methyltransferase complex. It appears to play a role in both transcriptional and post-transcriptional regulation of certain cellular genes and affect m6A methyltransferase activity (Little et al., 2000; Ping et al., 2014). Its involvement in regulating cell cycle, alternative splicing and cell proliferation have been suggested as well (Horiuchi et al., 2006; Small et al., 2006; Small and Pickering, 2009).

In addition, WTAP has been found to be a negative regulator of tumor suppressor protein Wilm’s tumor gene1(WT1), interacting with WT1 and inhibiting its transcription factor activity (Little et al., 2000). The involvement of WTAP in malignant tumors has been investigated. For example, WTAP was identified as a prognostic factor in glioblastoma and might be an oncogenic protein in acute myeloid leukemia (Di et al., 2012; Bansal et al., 2014). Over expression of WTAP promotes tumor growth and progression in hepatocellular carcinoma *via* HuR-ETS1 axis (Chen et al., 2019). WTAP can also promote invasion and metastasis of cancer cells by regulating epidermal growth factor receptor (EGFR) or mRNA expression (Di et al., 2012; Jo et al., 2013; Li et al., 2019; Li et al., 2022). Besides, the Warburg effect of cancers could be accelerated by WTAP through m6A-dependent regulation of hexokinase 2 (HK2) stability (Yu et al., 2021; Lyu et al., 2022). However, some studies suggest that WTAP may sometimes play a protective role. WTAP were down-regulated in lung adenocarcinoma, (Li et al., 2020b), and it was a protective gene in cutaneous melanoma prognosis (Feng et al., 2021). In addition, WTAP was essential for the tumor-suppressor function of carbonic anhydrase 4 (CA4) in colon cancer (Zhang et al., 2016). Upregulated WTAP expression was associated with less lymph node metastasis in breast cancer (Wang et al., 2022). In summary, WTAP may have opposite effect in different cancers, and the reason behind it remained elusive.

Our study further explores the role of WTAP in pan-cancer. Analysis of TCGA data in TIMER revealed that compared with normal tissues, the expression level of WTAP in tumor tissues vary in different cancers (Figure 1). In nine datasets in PrognoScan, high WTAP expression level could be used as a protective factor in brain, breast, colorectal and eye cancers, while a detrimental factor in lung cancer and ovarian cancer (Figure 2). In the Kaplan-Meier Plotter, high WTAP expression was found to be associated with good prognosis for BC, ESCC, KIRC, and READ, and with poor prognosis for CSCC, LIHC and OVC (Figure 3). This may challenge the previous finding of WTAP with shorter OS in esophageal cancer (Xu et al., 2020). Interestingly, the prognostic effect of WTAP on cancers was roughly consistent in different databases, and the inconsistencies in some may be due to the heterogeneity of data in the database and the potential influence of different biological characteristics. Further analysis of Kaplan-Meier Plotter showed that WTAP plays a detrimental role for LIHC patients, and the effects may vary in different genders, races, alcohol consumption, hepatitis infection, tumor grade, tumor stage and vascular invasion. It is worth noting that elevated WTAP has a particularly large effect on the prognosis of STAGE1 and 2, grade2 and 3 LIHC patients without vascular invasion, which may indicate that WTAP has a more significant effect on the early stages of cancer than on advanced cancer (Figure 4). Our findings strongly suggest that WTAP can be used as a prognostic biomarker for pan-cancer. In addition, the association between WTAP expression and prognosis varies across cancer types, suggesting that the function of WTAP in cancer may be multi-dimensional and complex.

Another finding is that WTAP levels are associated with diverse immune infiltration of immune cells. We explore the relationship between WTAP expression and the levels of numerous immune cells in LIHC and ESCA. As shown in Figure 5, the immune infiltration on macrophage and neutrophil were different in two types of cancer. After adjustment for tumor purity, in LIHC, macrophage and neutrophil were strongly correlated with WTAP expression while in ESCA these two immune cell types were less significant, indicating the promoting effect of WTAP on cancer may be dependent on macrophages and neutrophils (Table 1). In summary, these findings suggests that WTAP may influence patient survival *via* the recruitment and regulation of immune cells. Besides, recent studies shed light on the role of WTAP in the TME. Hypoxia in TME positively regulate increased expression of WTAP, which could promote glycolytic capacity, especially lactate production and extracellular acidification rate (Yu et al., 2021; Lyu et al., 2022). And lactate accumulation imbalance the lactate homeostasis in TME, promote tumor growth by suppressing immune cell activity and enhancing the immune escape of cancer cells (Gao et al., 2022; Pouysségur et al., 2022).

Previous studies also provide evidence of the contribution of immune cells in tumors. The tumor promoting functions of macrophages include supporting tumor-associated angiogenesis, promotion of tumor cell invasion, migration and intravasation as well as suppression of antitumor immune responses (Qian and Pollard, 2010). Other studies also suggested that neutrophil extracellular traps (NETs) promotes cancer metastasis *via* coiled-coil domain containing 25 (CCDC25) (Yang et al., 2020). In addition, higher neutrophil infiltration and increased macrophage density are reported to be associated with lower OS in hepatocellular carcinoma (Zhu et al., 2008; Schoenberg et al., 2021). These studies of the effect of immune cells in tumor may help to illustrate the findings from this study that macrophages and neutrophil have a positive correlation with WTAP expression level in LIHC and the high WTAP expression is associated with worse LIHC prognosis. Compared with ESCA, WTAP expression level in LIHC was strongly correlated with gene markers of most immune cells (Table 1), which further suggested that WTAP might promote cancer progression and influence patient survival through immune infiltration. But at the same time, we found that the high expression of WTAP also promoted the high infiltration of some immune cells that are thought to mediate the anti-tumor response, such as CD8+T cell and CD4+T cell (Cachot et al., 2021; Philip and Schietinger, 2022). It is hard to explain the contradictory results of these immune cell infiltration and cancer prognosis. Further studies exploring direct interactions at the cellular and molecular levels are needed. In addition to the multiple and complex roles of immune cells themselves, we speculate that the effect of WTAP on tumor progression and patient prognosis is complex and diverse rather than just immune effect.

In this article, we demonstrate that WTAP is closely associated with cancer, providing new potential biomarkers and therapeutic targets for pan-cancer treatment and prognosis assessment. However, there are limitations in our research. Firstly, the databases only focus on WTAP itself, but fail to reflect the information after translation and post-translational, while the post-translation modifications (PTMs) itself is quite diverse and complex. Secondly, we only conducted bioinformatics analysis of WTAP expression and patient survival rate in different databases, without *in vivo*/*in vitro* experiments, and failed to further clarify the specific mechanism of WTAP. Furtherly, although WTAP expression was found to be correlated with immune cell infiltration and patient prognosis, a cause-effect relationship could not be established and it could not explain or prove the relationship between immune infiltration and cancer survival.

In conclusion, the regulation of WTAP on cancer development deserves further exploration, and the specific molecular mechanism of WTAP action needs to be further elucidated in order to achieve its therapeutic potential in cancer and other diseases.

## Data Availability

The original contributions presented in the study are included in the article/Supplementary Material, further inquiries can be directed to the corresponding authors.
