# Supplementary material for: Comprehensive analysis about prognostic and immunological role of WTAP in pan-cancer
Source: Front Genet. 2022 Sep 6;13:1007696. doi: 10.3389/fgene.2022.1007696 (PMC9511574; doi:10.3389/fgene.2022.1007696)
Supplement: Supplementary file 1 [file DataSheet1.PDF]

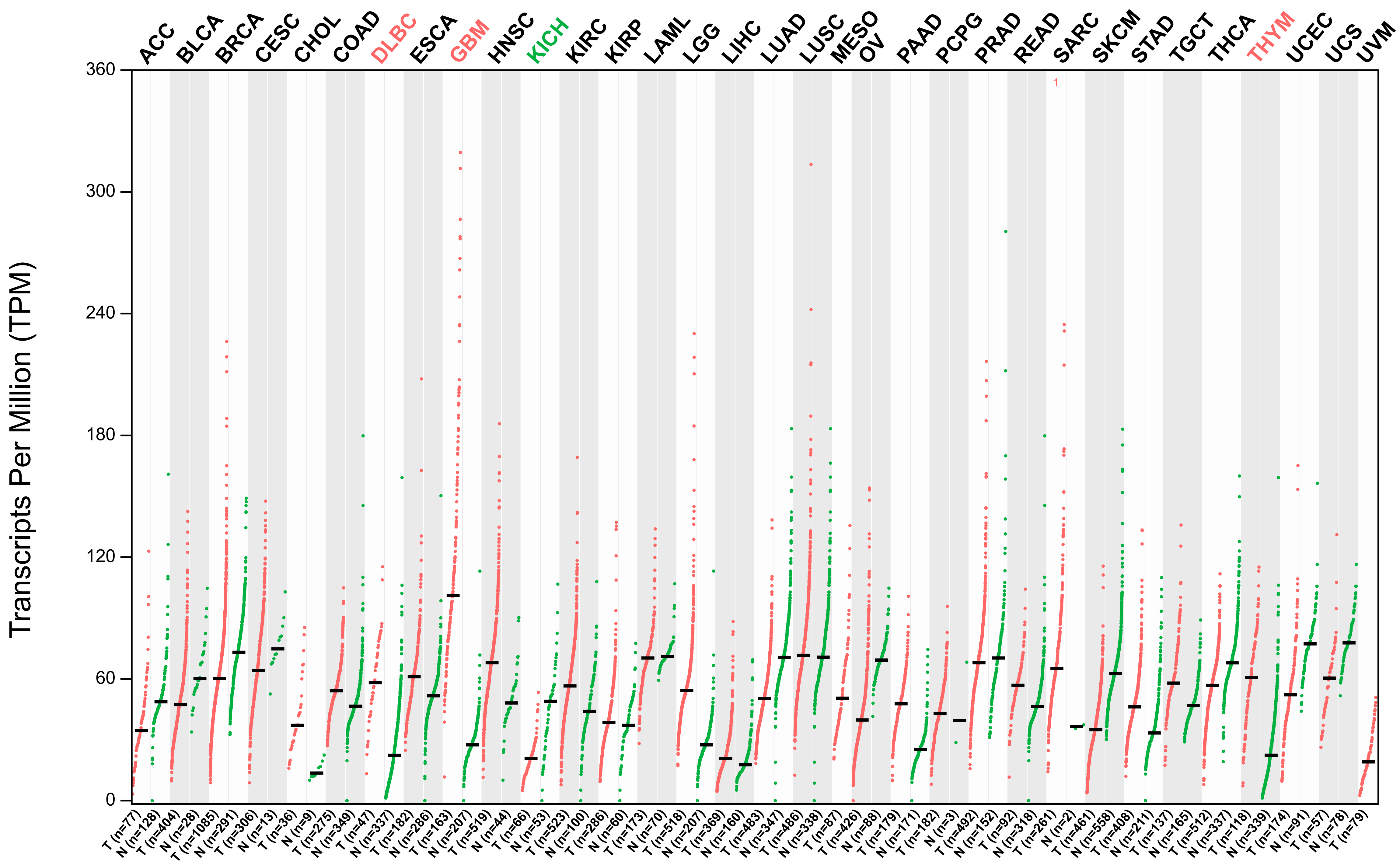

Transcripts Per Million (TPM)
ACC
BLCA
BRCA
CESC
CHOL
COAD
DLBC
ESCA
GBM
HNSC
KICH
KIRC
KIRP
LAML
LGG
LIHC
LUAD
LUSC
MESO
OV
PAAD
PCPG
PRAD
READ
SARC
SKCM
STAD
TGCT
THCA
THYM
UCEC
UCS
UVM
360
300
240
180
120
60
0
T (n=77)
N (n=128)
T (n=404)
N (n=28)
T (n=1085)
N (n=291)
T (n=306)
N (n=13)
T (n=36)
N (n=9)
T (n=275)
N (n=349)
T (n=47)
N (n=337)
T (n=182)
N (n=286)
T (n=163)
N (n=207)
T (n=519)
N (n=44)
T (n=66)
N (n=53)
T (n=523)
N (n=100)
T (n=286)
N (n=60)
T (n=173)
N (n=70)
T (n=518)
N (n=207)
T (n=369)
N (n=160)
T (n=483)
N (n=347)
T (n=486)
N (n=338)
T (n=87)
T (n=426)
N (n=88)
T (n=179)
N (n=171)
T (n=182)
N (n=3)
T (n=492)
N (n=152)
T (n=92)
N (n=318)
T (n=261)
N (n=2)
T (n=461)
N (n=558)
T (n=408)
N (n=211)
T (n=137)
N (n=165)
T (n=512)
N (n=337)
T (n=118)
N (n=339)
T (n=174)
N (n=91)
T (n=57)
N (n=78)
T (n=79)

Supplementary Figure 1 | The WTAP expression profile across tumor samples and paired normal tissues in GEPIA. Red dots stand for tumor samples while green dots stand for normal samples. ACC Adrenocortical carcinoma. |Log2FC| cutoff is set as 1, q cutoff is set as 0.1.
